# The management of acute coronary syndromes in patients presenting without persistent ST-segment elevation: early invasive strategy for all?

**DOI:** 10.1007/s12471-016-0944-1

**Published:** 2017-01-05

**Authors:** F. Arslan, M. Voskuil

**Affiliations:** 1 Laboratory of Experimental Cardiology, University Medical Center Utrecht, Utrecht, The Netherlands; 2 Department of Cardiology, University Medical Center Utrecht, Utrecht, The Netherlands

It is unquestionable that early invasive reperfusion therapy in the setting of ST-segment elevation myocardial infarction (STEMI) has significantly reduced infarct-related mortality and morbidity [1]. Scientific and social dissemination campaigns have created a certain awareness both in the public domain and among physicians with slogans such as ‘the sooner the better’ and ‘time is muscle’, when faced with such acute situations. The early diagnosis of ischaemia plays an equally important role in improving outcome after myocardial infarction (MI). Cardiac troponins (cTn) play a central role in detecting myocardial damage and are regarded as a cornerstone in the diagnosis of an acute coronary event resulting from atherosclerotic plaque instability [2]. On intracoronary imaging studies and post-mortem observation, acute coronary syndrome without persistent ST-segment elevation (i. e. non-STEMI, NSTEMI-ACS) is often seen to be related to ‘milder’ forms of plaque instability such as erosion and ulceration-based instability [3–5]. The high-sensitive cTn assays have put the definition of MI into a new perspective [2]. Early detection is preferable in any type of injury or disease, but the question of whether earlier intervention will also result in favourable outcome at an acceptable cost remains.

In this issue of the *Netherlands Heart Journal*, Damman and colleagues give the Dutch ACS Working Group perspective on the 2015 ESC Guidelines for the management of acute coronary syndromes in patients presenting with NSTEMI.[6] It is a concise analysis which compares the most relevant changes with the 2011 guidelines for general cardiology practice. In this editorial, we would like to further elaborate on the same-day transfer policy in high-risk patients.

The current NSTEMI-ACS guidelines put more emphasis on the immediate transfer and invasive management on the same day and/or within 24 h of high-risk patients than the 2011 version [7]. Fig. 6 and Table 13 in the guidelines illustrate the timing and treatment strategy in accordance with a certain risk stratification. If a very-high-risk criterion is met, urgent invasive management is required. This, of course, is not under debate. However, if one out of the three high-risk criteria is met, same-day transfer is recommended as a Class I indication with level of evidence A (Section 5.6.9 of the guidelines). Unfortunately, the underlying evidence for such an indication and strategy is lacking, even in the literature that is used by the ESC committee to substantiate the recommendation.

The studies referred to in the 2015 guidelines are the TIMACS trial and two meta-analyses [8–10]. In the TIMACS trial, 3031 NSTEMI-ACS patients were randomly assigned to undergo either early (<24 h) or delayed (>36 h) coronary angiography after randomisation. There was no difference between early and delayed interventions in the primary outcome (composite of death, MI or stroke at 6 months). Patients undergoing early intervention experienced less refractory ischaemia at the cost of more repeat revascularisations 30 days after randomisation. A very interesting finding was seen in a prespecified subgroup analysis. It was only in patients with a GRACE risk score >140, and not biomarker rise-and-fall and dynamic ST-T segments as outlined in the ESC 2015 guidelines, that a significant reduction in the primary outcome was shown. The lack of benefit of early interventional strategies in preventing death and MI was also a conclusion of the meta-analyses referred to by the 2015 guidelines [8, 9].

More recent studies fail to show any hard clinical benefit or a benefit in survival in the early over delayed invasive strategy. The RIDDLE-NSTEMI study is a small randomised trial with 323 patients randomised to either immediate (<2 h) or delayed (2–72 h) intervention. Thirty days after the index event, 0 vs. 10 new MIs (pre-catheterisation) were observed in early vs. delayed strategy, respectively. However, there were more patients with a GRACE risk score >140 in the delayed group. Furthermore, CABG was the preferred revascularisation strategy in 24% of the delayed intervention patients, while in the early intervention group only 12% underwent CABG (*p* = 0.01). This indicates a higher risk profile in the delayed intervention group, despite randomisation [11].

The OPTIMA trial randomised 142 patients to immediate or deferred (24–48 h) PCI after a diagnostic angiography was performed within 3 h of admission. Deferral of PCI resulted in fewer MIs when compared with immediate intervention (38% vs. 60%, *p* = 0.005) at 30 days and 6 months [12]. This difference is rather remarkable given the fact that more patients in the deferred group had a TIMI flow of 0–2 (36% vs. 19%, *p* = 0.02). Five-year follow-up of the trial did not show any difference between the groups in the composite of death and spontaneous MI [13].

The ELISA trial randomised 542 patients to either early (<12 h) or delayed (>48 h) invasive strategy (angiography and revascularisation if appropriate). The study failed to show the superiority of early invasive management over a delayed strategy in terms of death, re-infarction or recurrent ischaemia 30 days after NSTEMI-ACS [14].

A recent analysis of 4307 NSTEMI-ACS patients from the Melbourne Interventional Group registry observed no mortality hazard in >24 h deferral of PCI during 12-month follow-up. The main predictors of mortality were older age, impaired renal function and elevated biomarkers which are highly relevant in the GRACE risk score.

So far, studies and meta-analysis have not shown that invasive strategy within 24 h of admission with NSTEMI-ACS confers any benefit. Detailed analysis of the data only justifies early invasive management in patients with a GRACE risk score >140. In the absence of MI-related complications or refractory angina despite optimal medical treatment, dynamic ST or T‑wave changes or a rise and fall compatible with MI appears to be an insufficient high-risk criterion for same-day transfer to a PCI centre. With the high-sensitive cTn assays in particular, there will be over-classification of high-risk patients. To corroborate the statements of Damman and colleagues, we believe that an early invasive strategy (<24 h) as a Class IA indication is not supported by the literature, and that it may only be justified in patients with a GRACE risk score >140. Same-day transfer of these ‘high-risk’ patients with NSTEMI to an intervention centre is not likely to result in a clear health benefit. Future studies have to be awaited to clarify early invasive strategy [15].
